# 2-Methyl-*N*-(3-methyl­phen­yl)benzamide

**DOI:** 10.1107/S1600536808003103

**Published:** 2008-01-30

**Authors:** B. Thimme Gowda, Sabine Foro, B. P. Sowmya, Hartmut Fuess

**Affiliations:** a Department of Chemistry, Mangalore University, Mangalagangotri 574 199, Mangalore, India; b Institute of Materials Science, Darmstadt University of Technology, Petersenstrasse 23, D-64287 Darmstadt, Germany

## Abstract

In the structure of the title compound (N3MP2MBA), C_15_H_15_NO, the conformation of the N—H bond is *anti* to the *meta*-methyl substituent in the aniline ring and that of the C=O bond is *syn* to the *ortho*-methyl substituent in the benzoyl ring, while the conformations of the N—H and C=O bonds are *anti* to each other. The bond parameters in N3MP2MBA are similar to those in 2-methyl-*N*-phenyl­benzamide, *N*-(3,4-dimethyl­phen­yl)benzamide and other benzanilides. The amide group, –NHCO–, makes a dihedral angle of 55.2 (7)° with the benzoyl ring, while the dihedral angle between the two benzene rings (benzoyl and aniline) is 36.2 (1)°. N—H⋯O hydrogen bonds give rise to infinite chains running along the *b* axis of the crystal structure.

## Related literature

For related literature, see: Gowda *et al.* (2003 ▶; 2008*a* ▶,*b* ▶).
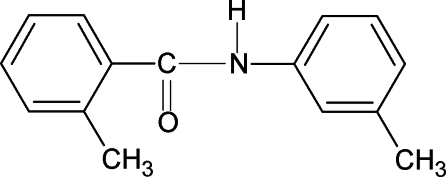


## Experimental

### 

#### Crystal data

C_15_H_15_NO
*M* *_r_* = 225.28
Tetragonal, 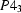

*a* = 8.931 (2) Å
*c* = 15.816 (4) Å
*V* = 1261.5 (3) Å^3^
*Z* = 4
Cu *K*α radiation
μ = 0.58 mm^−1^
*T* = 299 (2) K
0.55 × 0.30 × 0.30 mm

#### Data collection

Enraf–Nonius CAD-4 diffractometer
Absorption correction: none
1606 measured reflections
1168 independent reflections
1096 reflections with *I* > 2σ(*I*)
*R*_int_ = 0.016
3 standard reflections frequency: 120 min intensity decay: 1.0%

#### Refinement

*R*[*F* ^2^ > 2σ(*F* ^2^)] = 0.036
*wR*(*F* ^2^) = 0.100
*S* = 1.07
1168 reflections
158 parameters
2 restraints
H atoms treated by a mixture of independent and constrained refinement
Δρ_max_ = 0.12 e Å^−3^
Δρ_min_ = −0.10 e Å^−3^

### 

Data collection: *CAD-4-PC* (Enraf–Nonius, 1996 ▶); cell refinement: *CAD-4-PC*; data reduction: *REDU4* (Stoe & Cie, 1987 ▶); program(s) used to solve structure: *SHELXS97* (Sheldrick, 2008 ▶); program(s) used to refine structure: *SHELXL97* (Sheldrick, 2008 ▶); molecular graphics: *PLATON* (Spek, 2003 ▶); software used to prepare material for publication: *SHELXL97*.

## Supplementary Material

Crystal structure: contains datablocks I, global. DOI: 10.1107/S1600536808003103/om2212sup1.cif

Structure factors: contains datablocks I. DOI: 10.1107/S1600536808003103/om2212Isup2.hkl

Additional supplementary materials:  crystallographic information; 3D view; checkCIF report

## Figures and Tables

**Table 1:** Hydrogen-bond geometry (Å, °)

*D*—H⋯*A*	*D*—H	H⋯*A*	*D*⋯*A*	*D*—H⋯*A*
N1—H1*N*⋯O1^i^	0.837 (18)	2.10 (2)	2.908 (3)	163 (3)

Symmetry code: (i) 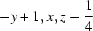.

**Fig. 1.**
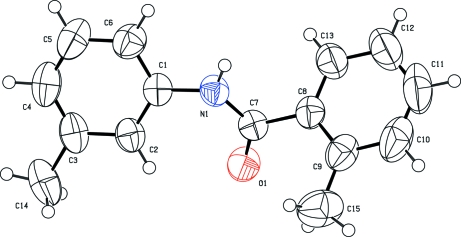
Molecular structure of the title compound, showing the atom labeling scheme. The displacement ellipsoids are drawn at the 50% probability level.

**Fig. 2.**
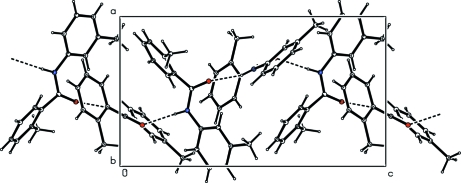
Molecular packing of the title compound with hydrogen bonding shown as dashed lines.

## supplementary crystallographic information

### Comment

As part of a study of the substituent effects on the structures of N-aromatic amides, in the present work, the structure of *N*-(3-methylphenyl)-2-methylbenzamide (N3MP2MBA) has been determined (Gowda *et al.*, 2003; 2008*a*; 2008*b*). In the structure of N3MP2MBA (Fig. 1), the conformation of the N—H bond is *anti* to the *meta*-methyl substituent in the aniline ring and that of the C=O bond is *syn* to the *ortho*-methyl substituent in the benzoyl ring, while the conformations of the N—H and C=O bonds are *anti* to each other. The bond parameters in N2MP2MBA are similar to those in *N*-(phenyl)-2-methylbenzamide (Gowda *et al.*, 2008*a*), *N*-(3,4-dimethylphenyl)-benzamide (Gowda *et al.*, 2008*b*) and other benzanilides (Gowda *et al.*, 2003). The amide group –NHCO– has the dihedral angle of 55.2 (7)° with the benzoyl ring, while the dihedral angle between the two benzene rings (benzoyl and aniline) is 36.2 (1)°. The packing diagram of N3MP2MBA molecules showing the hydrogen bonds N1—H1N···O1 (Table 1) involved in the formation of molecular chain is given in Fig. 2.

### Experimental

The title compound was prepared according to the literature method (Gowda *et al.*, 2003). The purity of the compound was checked by determining its melting point. It was characterized by recording its infrared and NMR spectra. Single crystals of the title compound were obtained from an ethanolic solution and used for X-ray diffraction studies at room temperature.

### Refinement

The NH atom was located in difference map and was refined with restrained geometry, *viz*. N—H distance was restrained to 0.86 (2) Å. The other H atoms were positioned with idealized geometry using a riding model with C—H = 0.93–0.96 Å A l l H atoms were refined with isotropic displacement parameters (set to 1.2 times of the *U*_eq_ of the parent atom).

In the absence of significant anomalous dispersion effects, Friedel pairs were merged and the Δf"term set to zero.

### Crystal data

**Table:**

C_15_H_15_NO	*Z* = 4
*M**_r_* = 225.28	*F*_000_ = 480
Tetragonal, *P*4_3_	*D*_x_ = 1.186 Mg m^−^^3^
Hall symbol: P 4cw	Cu *K*α radiation λ = 1.54180 Å
*a* = 8.931 (2) Å	Cell parameters from 25 reflections
*b* = 8.931 (2) Å	θ = 4.9–19.0º
*c* = 15.816 (4) Å	µ = 0.58 mm^−^^1^
α = 90º	*T* = 299 (2) K
β = 90º	Prism, colourless
γ = 90º	0.55 × 0.30 × 0.30 mm
*V* = 1261.5 (3) Å^3^	

### Data collection

**Table:**

Enraf–Nonius CAD-4 diffractometer	*R*_int_ = 0.016
Radiation source: fine-focus sealed tube	θ_max_ = 66.8º
Monochromator: graphite	θ_min_ = 5.0º
*T* = 299(2) K	*h* = −10→0
ω/2θ scans	*k* = −10→0
Absorption correction: none	*l* = −18→3
1606 measured reflections	3 standard reflections
1168 independent reflections	every 120 min
1096 reflections with *I* > 2σ(*I*)	intensity decay: 1.0%

### Refinement

**Table:**

Refinement on *F*^2^	Secondary atom site location: difference Fourier map
Least-squares matrix: full	Hydrogen site location: inferred from neighbouring sites
*R*[*F*^2^ > 2σ(*F*^2^)] = 0.036	H atoms treated by a mixture of independent and constrained refinement
*wR*(*F*^2^) = 0.100	*w* = 1/[σ^2^(*F*_o_^2^) + (0.0637*P*)^2^ + 0.0805*P*] where *P* = (*F*_o_^2^ + 2*F*_c_^2^)/3
*S* = 1.07	(Δ/σ)_max_ < 0.001
1168 reflections	Δρ_max_ = 0.12 e Å^−^^3^
158 parameters	Δρ_min_ = −0.10 e Å^−^^3^
2 restraints	Extinction correction: SHELXL97 (Sheldrick, 2008), Fc^*^=kFc[1+0.001xFc^2^λ^3^/sin(2θ)]^-1/4^
Primary atom site location: structure-invariant direct methods	Extinction coefficient: 0.0069 (14)

### Special details

**Table:**

Geometry. All e.s.d.'s (except the e.s.d. in the dihedral angle between two l.s. planes) are estimated using the full covariance matrix. The cell e.s.d.'s are taken into account individually in the estimation of e.s.d.'s in distances, angles and torsion angles; correlations between e.s.d.'s in cell parameters are only used when they are defined by crystal symmetry. An approximate (isotropic) treatment of cell e.s.d.'s is used for estimating e.s.d.'s involving l.s. planes.
Refinement. Refinement of *F*^2^ against ALL reflections. The weighted *R*-factor *wR* and goodness of fit *S* are based on *F*^2^, conventional *R*-factors *R* are based on *F*, with *F* set to zero for negative *F*^2^. The threshold expression of *F*^2^ > σ(*F*^2^) is used only for calculating *R*-factors(gt) *etc*. and is not relevant to the choice of reflections for refinement. *R*-factors based on *F*^2^ are statistically about twice as large as those based on *F*, and *R*- factors based on ALL data will be even larger.

### Fractional atomic coordinates and isotropic or equivalent isotropic displacement parameters (Å^2^)

**Table:**

	*x*	*y*	*z*	*U*_iso_*/*U*_eq_	
C1	0.3124 (3)	0.2503 (2)	0.05561 (14)	0.0534 (5)	
C2	0.2079 (3)	0.2607 (3)	0.12012 (15)	0.0601 (6)	
H2	0.1629	0.3523	0.1317	0.072*	
C3	0.1696 (3)	0.1353 (3)	0.16775 (15)	0.0681 (7)	
C4	0.2385 (4)	0.0013 (3)	0.1499 (2)	0.0827 (9)	
H4	0.2142	−0.0833	0.1813	0.099*	
C5	0.3424 (4)	−0.0095 (3)	0.0865 (2)	0.0874 (9)	
H5	0.3877	−0.1011	0.0752	0.105*	
C6	0.3802 (3)	0.1149 (3)	0.03921 (17)	0.0718 (7)	
H6	0.4512	0.1072	−0.0036	0.086*	
C7	0.3347 (2)	0.5195 (2)	0.01949 (14)	0.0521 (5)	
C8	0.3969 (3)	0.6215 (3)	−0.04697 (15)	0.0576 (6)	
C9	0.3085 (4)	0.7335 (3)	−0.08194 (17)	0.0729 (7)	
C10	0.3766 (6)	0.8318 (4)	−0.1386 (2)	0.1034 (12)	
H10	0.3200	0.9078	−0.1630	0.124*	
C11	0.5257 (7)	0.8189 (4)	−0.1590 (3)	0.1172 (16)	
H11A	0.5692	0.8874	−0.1958	0.141*	
C12	0.6101 (5)	0.7064 (5)	−0.1257 (3)	0.1091 (13)	
H12A	0.7102	0.6964	−0.1408	0.131*	
C13	0.5459 (4)	0.6074 (3)	−0.0694 (2)	0.0774 (8)	
H13	0.6033	0.5307	−0.0463	0.093*	
C14	0.0560 (4)	0.1486 (5)	0.2363 (2)	0.0956 (10)	
H14A	−0.0376	0.1807	0.2127	0.115*	
H14B	0.0894	0.2206	0.2773	0.115*	
H14C	0.0431	0.0531	0.2632	0.115*	
C15	0.1464 (4)	0.7517 (5)	−0.0604 (3)	0.1053 (12)	
H15A	0.1361	0.7678	−0.0007	0.126*	
H15B	0.0928	0.6629	−0.0763	0.126*	
H15C	0.1064	0.8361	−0.0904	0.126*	
N1	0.3505 (2)	0.3731 (2)	0.00353 (13)	0.0561 (5)	
H1N	0.392 (3)	0.350 (3)	−0.0422 (14)	0.067*	
O1	0.2763 (2)	0.56893 (19)	0.08395 (11)	0.0681 (5)	

### Atomic displacement parameters (Å^2^)

**Table:**

	*U*^11^	*U*^22^	*U*^33^	*U*^12^	*U*^13^	*U*^23^
C1	0.0613 (12)	0.0547 (12)	0.0444 (11)	−0.0074 (9)	−0.0062 (10)	−0.0018 (9)
C2	0.0662 (13)	0.0625 (13)	0.0515 (13)	−0.0094 (10)	−0.0007 (11)	0.0011 (11)
C3	0.0790 (16)	0.0749 (16)	0.0504 (14)	−0.0251 (13)	−0.0092 (12)	0.0083 (12)
C4	0.112 (2)	0.0683 (16)	0.0680 (17)	−0.0192 (15)	−0.0136 (17)	0.0189 (14)
C5	0.123 (2)	0.0562 (14)	0.083 (2)	0.0044 (15)	−0.007 (2)	0.0070 (15)
C6	0.0897 (18)	0.0639 (14)	0.0619 (16)	0.0042 (12)	0.0023 (14)	−0.0021 (12)
C7	0.0558 (11)	0.0558 (11)	0.0447 (11)	−0.0033 (9)	−0.0023 (9)	−0.0026 (9)
C8	0.0721 (14)	0.0517 (12)	0.0490 (12)	−0.0063 (10)	0.0031 (11)	−0.0049 (10)
C9	0.102 (2)	0.0610 (14)	0.0560 (15)	0.0059 (13)	−0.0007 (14)	0.0014 (12)
C10	0.173 (4)	0.0676 (17)	0.070 (2)	0.008 (2)	0.016 (2)	0.0166 (15)
C11	0.180 (4)	0.078 (2)	0.093 (3)	−0.043 (3)	0.047 (3)	0.003 (2)
C12	0.111 (3)	0.093 (2)	0.123 (3)	−0.033 (2)	0.047 (3)	−0.007 (2)
C13	0.0777 (17)	0.0716 (16)	0.0828 (19)	−0.0131 (13)	0.0141 (15)	−0.0023 (14)
C14	0.107 (2)	0.112 (2)	0.0678 (18)	−0.0414 (19)	0.0137 (18)	0.0095 (18)
C15	0.105 (3)	0.125 (3)	0.086 (2)	0.038 (2)	−0.011 (2)	0.012 (2)
N1	0.0679 (12)	0.0564 (10)	0.0440 (9)	−0.0042 (8)	0.0088 (9)	−0.0027 (8)
O1	0.0946 (12)	0.0628 (10)	0.0468 (9)	0.0015 (9)	0.0096 (9)	−0.0054 (8)

### Geometric parameters (Å, °)

**Table:**

C1—C6	1.377 (4)	C9—C10	1.394 (5)
C1—C2	1.386 (3)	C9—C15	1.496 (5)
C1—N1	1.414 (3)	C10—C11	1.375 (7)
C2—C3	1.392 (3)	C10—H10	0.9300
C2—H2	0.9300	C11—C12	1.361 (7)
C3—C4	1.375 (5)	C11—H11A	0.9300
C3—C14	1.490 (5)	C12—C13	1.380 (5)
C4—C5	1.369 (5)	C12—H12A	0.9300
C4—H4	0.9300	C13—H13	0.9300
C5—C6	1.381 (4)	C14—H14A	0.9600
C5—H5	0.9300	C14—H14B	0.9600
C6—H6	0.9300	C14—H14C	0.9600
C7—O1	1.227 (3)	C15—H15A	0.9600
C7—N1	1.339 (3)	C15—H15B	0.9600
C7—C8	1.498 (3)	C15—H15C	0.9600
C8—C13	1.382 (4)	N1—H1N	0.837 (18)
C8—C9	1.389 (4)		
			
C6—C1—C2	119.6 (2)	C11—C10—C9	121.4 (4)
C6—C1—N1	117.8 (2)	C11—C10—H10	119.3
C2—C1—N1	122.6 (2)	C9—C10—H10	119.3
C1—C2—C3	120.6 (2)	C12—C11—C10	120.5 (3)
C1—C2—H2	119.7	C12—C11—H11A	119.8
C3—C2—H2	119.7	C10—C11—H11A	119.8
C4—C3—C2	118.6 (3)	C11—C12—C13	119.5 (4)
C4—C3—C14	121.6 (3)	C11—C12—H12A	120.2
C2—C3—C14	119.8 (3)	C13—C12—H12A	120.2
C5—C4—C3	121.0 (3)	C12—C13—C8	120.4 (3)
C5—C4—H4	119.5	C12—C13—H13	119.8
C3—C4—H4	119.5	C8—C13—H13	119.8
C4—C5—C6	120.4 (3)	C3—C14—H14A	109.5
C4—C5—H5	119.8	C3—C14—H14B	109.5
C6—C5—H5	119.8	H14A—C14—H14B	109.5
C1—C6—C5	119.8 (3)	C3—C14—H14C	109.5
C1—C6—H6	120.1	H14A—C14—H14C	109.5
C5—C6—H6	120.1	H14B—C14—H14C	109.5
O1—C7—N1	123.5 (2)	C9—C15—H15A	109.5
O1—C7—C8	121.5 (2)	C9—C15—H15B	109.5
N1—C7—C8	114.97 (19)	H15A—C15—H15B	109.5
C13—C8—C9	120.7 (3)	C9—C15—H15C	109.5
C13—C8—C7	118.8 (2)	H15A—C15—H15C	109.5
C9—C8—C7	120.4 (2)	H15B—C15—H15C	109.5
C8—C9—C10	117.5 (3)	C7—N1—C1	128.5 (2)
C8—C9—C15	122.5 (3)	C7—N1—H1N	117 (2)
C10—C9—C15	120.0 (3)	C1—N1—H1N	115 (2)
			
C6—C1—C2—C3	−0.7 (3)	C7—C8—C9—C10	175.1 (3)
N1—C1—C2—C3	177.5 (2)	C13—C8—C9—C15	179.2 (3)
C1—C2—C3—C4	0.5 (4)	C7—C8—C9—C15	−4.4 (4)
C1—C2—C3—C14	−179.5 (3)	C8—C9—C10—C11	−0.1 (5)
C2—C3—C4—C5	−0.2 (4)	C15—C9—C10—C11	179.4 (4)
C14—C3—C4—C5	179.8 (3)	C9—C10—C11—C12	1.7 (6)
C3—C4—C5—C6	0.1 (5)	C10—C11—C12—C13	−1.7 (7)
C2—C1—C6—C5	0.7 (4)	C11—C12—C13—C8	0.3 (6)
N1—C1—C6—C5	−177.7 (3)	C9—C8—C13—C12	1.3 (5)
C4—C5—C6—C1	−0.4 (5)	C7—C8—C13—C12	−175.2 (3)
O1—C7—C8—C13	122.6 (3)	O1—C7—N1—C1	−3.0 (4)
N1—C7—C8—C13	−56.2 (3)	C8—C7—N1—C1	175.7 (2)
O1—C7—C8—C9	−53.9 (3)	C6—C1—N1—C7	−159.7 (2)
N1—C7—C8—C9	127.4 (2)	C2—C1—N1—C7	22.0 (4)
C13—C8—C9—C10	−1.3 (4)		

### Hydrogen-bond geometry (Å, °)

**Table:**

*D*—H···*A*	*D*—H	H···*A*	*D*···*A*	*D*—H···*A*
N1—H1N···O1^i^	0.837 (18)	2.10 (2)	2.908 (3)	163 (3)

Symmetry codes: (i) −*y*+1, *x*, *z*−1/4.
